# Challenges and Strategies in Implementing Hospital Accreditation Standards Among Healthcare Professionals in Healthcare Systems in Yemen: A Phenomenological Study

**DOI:** 10.7759/cureus.59383

**Published:** 2024-04-30

**Authors:** Talal Mansoor, Sharifa Ezat Wan Puteh, Azimatun Noor Aizuddin, Malakeh Z Malak

**Affiliations:** 1 Department of Community Health, Faculty of Medicine, Universiti Kebangsaan Malaysia Medical Centre, Kuala Lumpur, MYS; 2 Department of Public Health Medicine, Faculty of Medicine, Universiti Kebangsaan Malaysia Medical Centre, Kuala Lumpur, MYS; 3 Faculty of Nursing, Al-Zaytoonahh University of Jordan, Amman, JOR

**Keywords:** hospital accreditation, healthcare systems, yemen, strategies, implementation, challenges, accreditation standards

## Abstract

Introduction: The implementation of hospital accreditation standards in healthcare systems in Yemen that ensure safe and high-quality healthcare services is hampered by specific challenges. Therefore, this study was purposed to explore the challenges and strategies for applying hospital accreditation standards among healthcare professionals in Yemen.

Methods: A qualitative, phenomenological design was adopted to conduct this study. Semi-structured interviews were used to collect data during the period from January 1, 2022, to February 28, 2022.

Results: Based on the content analysis, the study outcomes and lack of (i) funding, (ii) competent human resources, (iii) optimal infrastructure, and (iv) equipment and supplies deter the implementation of hospital accreditation standards. Also, this study highlighted the cultural and social barriers limiting the effectiveness of hospital accreditation standards, the need for increased investment in healthcare infrastructure and human resources, and cultural sensitivity training for healthcare professionals to enhance the implementation of and compliance with hospital accreditation standards.

Conclusions: Policymakers should engage global corporations and development partners for technical assistance and capacity building that support the local application of hospital accreditation standards.

## Introduction

The World Health Organization (WHO) reported that the health system in Yemen is fragile due to poor staff morale and services and various structural and service delivery issues [1]. This notable lag in healthcare standards following global, economic, and political crises has caused Yemen to fall behind other developing nations. Alternatively, adopting a Western model potentially optimizes the current healthcare standards in Yemen.

The International Society for Quality in Healthcare (ISQua) defined accreditation as a public recognition by a healthcare accreditation body of the achievement of accreditation standards by a healthcare organization, demonstrated through an independent external peer assessment of that organization’s level of performance about the standards [2]. Essentially, accreditation serves to enhance patients’ anticipated treatment outcomes and the quality of healthcare services. In terms of professional development, accreditation in nursing promotes effective practices that improve the quality of care, increase public trust, leadership, and accountability, and establish a safe and conducive environment [3]. Developed countries have popularised the notion of accreditation among their developing counterparts in the past three decades, thus globally increasing the number of accreditation programs [4].

Previous studies proposed that many challenges impede the application of accreditation and quality systems. For example, Kakemam et al. claimed that a lack of managerial support, visibility, and training and education deterred employees from engaging in quality improvement and following up on accreditation survey outcomes [5]. They also revealed that poor cultural infrastructure, insufficient finances, and hampered the implementation of accreditation.

From the perspective of hospital nurses, a high workload results from implementing accreditation given the lack of employees allotted to the task. Ng et al. outlined that low awareness of quality enhancement, lack of applicable accreditation standards, insufficient performance measures, high staff workload, and organizational resistance to change were factors hindering the implementation of accreditation in hospitals [6]. In Iranian hospitals, poor program design, hospital deficiencies, fundamental drawbacks in the local healthcare systems, surveyors’ difficulties with the survey process, high staff workload, and the adverse implications of the hospital program deterred the inclusion of accreditation [7].

Likewise, a lack of manpower, low-quality infrastructure, and insufficient financial resources hampered the implementation of hospital accreditation [8]. Low- and middle-income countries (LMICs) struggle to get accreditation for hospitals following inadequate technology levels and hospital infrastructure [8]. Hospital accreditation positively impacts the quality of care, albeit with drawbacks involving insufficient financial and capital resources and staff-, organization-, and patient-related limitations [9,10].

It is deemed challenging to alter environmental management practices in hospitals given the lack of awareness on the subject matter [10]. According to Mosadeghrad, inadequate resources can adversely affect the quality of healthcare services [11]. Yousefinezhadi et al. argued that efficient accreditation programs rely on multiple stakeholders’ engagement, the number of resources (including human resources), and the availability of sustainable funds [12]. Training and educating hospital managers, providing the necessary resources and incentives to implement standards, determining a specific time to apply the standards, and pilot-testing the standards before applying the accreditation to hospitals are integral to implementing accreditation in hospitals. In other words, hospital employees and managers who disregard these factors would find the accreditation process burdensome [13]. Bahmaei et al. underscored the importance of optimally training the hospital staff for the implementation, allocating a specific budget for the program, and setting and complying with performance indicators [14].

Literature explicitly indicates the barriers to implementing accreditation in healthcare systems. This knowledge is necessary for healthcare systems to develop policies and measures that ensure accreditation and quality. However, there is a lack of research in this area in Yemen; therefore, this study could help policymakers understand healthcare professionals' perspectives on challenges and strategies to implement accreditation and quality in healthcare systems.

This qualitative study aimed to explore the Yemini healthcare professionals’ experiences regarding challenges and strategies in the implementation of accreditation and quality standards in a collectivist society, with extensive discussions on data derived from the study participants. Notably, the current work was guided by the following question: “What are the challenges and strategies in implementing accreditation and quality systems and associated countermeasures in the hospitals of the Hadhramaut region?"

## Materials and methods

Study design

This study employed a descriptive phenomenological method by Husserl [15], where a phenomenon should be conveyed through individual voices. According to Husserl’s philosophy [16], researchers must disregard past knowledge, pre-conceived notions, and judgments about the phenomenon under study pre-data collection to avoid misinterpreting the outcomes [17]. Phenomenological scholars who gather and evaluate qualitative data should practice phenomenological reduction by posing specific questions to themselves. The study was approved by the Institutional Review Board at the University Kebangsaan Malaysia (approval number: NO# UKM PPI/111/8/JEP-2020-175).

Sample and setting

Professionals who are highly experienced with quality care and quality systems were invited to share their experiences, which provides empirical data [18]. The study population constituted all healthcare professionals from major hospitals in the Hadhramaut region of Yemen with expertise in quality systems. Only healthcare professionals specializing in quality systems at least a year before data collection and willing to participate were included in this study. The sample size depended on data saturation, which was attained on the 10th participant, as the collected data revealed no new findings [19]. Ten healthcare professionals with quality-related experience were selected via purposive sampling given its propriety in providing data that addresses the research question [20].

Data collection

Data were gathered through semi-structured interviews, which were conducted in person during the study period from January 1, 2022, to February 28, 2022. These semi-structured interviews with pre-determined open-ended questions were conducted at a time convenient to the researcher and participants. Participants were informed that the interviews would be recorded to facilitate data gathering and ensure data quality. They were asked to share their experiences with quality systems in their own words. Each interview was then transcribed verbatim. Furthermore, the transcribed interviews were reviewed and rechecked parallel to the recorded materials. Each participant was labeled with a code in place of personal information to maintain confidentiality.

Data analysis

Data collection and analysis were performed simultaneously to elicit enriched data. Specifically, the data were analyzed manually with Colaizzi’s seven-step phenomenological data analysis framework [21]: (i) familiarization, where the researchers internalized data upon reading each transcribed interview several times; (ii) identification of key statements, in which all statements in the narrative directly relating to quality systems were determined; (iii) the formulation of meanings, where meaning associated with the phenomenon of interest was concluded by emphasizing significant statements; (iv) the thematical arrangement of key statements and determined meanings across all the interviews; (v) the creation of an exhaustive description, in which a comprehensive description of the phenomenon of interest was written with all the themes involved; (vi) the consolidation of the exhaustive description into a short and intense statement, which is integral to the phenomenon and presents the fundamental structure; and (vii) outcome validation by the participants. The themes were outlined through relevant quotes.

## Results

Participant characteristics

This study selected 10 healthcare professionals between 30 and 50 years of age; all were male. The participants’ experience in quality systems ranged between two and 15 years. Four themes on the challenges in accreditation and quality systems and two on strategies for quality standards application were derived post-interview analysis.

Challenges in implementing accreditation and quality systems

Themes on the issues underlying accreditation involved lack of proper infrastructure, financial constraints, lack of awareness of the quality system and accreditation, and lack of monitoring of healthcare professionals. Table 1 summarizes these themes with sub-themes and quotations.

**Table 1:** Challenges in implementing accreditation and quality systems O, A1, F, M, A2, A3, H, N, B indicate the different participants

Theme	Sub-theme	Supporting quotation
Lack of proper infrastructure	Lack of proper medical equipment.	“The obstacles facing hospitals are many, such as lack of proper medical equipment medical staff. For example, the medical devices in many of our healthcare centers are outdated when compared with those in other countries”. [O]
	Inadequately trained medical staff.	“The Health Office has offered some support in Hadhramaut hospitals but it’s inadequate for many hospitals, especially those in rural areas. Continuous medical staff training and development are requested but not provided”. [A1]
	Absence of a management approach.	“Infrastructure is very important and without it, we lack quality. Thus, constructing a high-quality infrastructure is necessary to support the healthcare quality system. The main problem in our hospitals is that the managers are not focused on supplying their hospitals with technology and high-quality devices and tools”. [F].
	Lack of novel quality systems and poor application of quality standards	“Quality standards in Yemen are not applied consistently at all, for example, if any medical device is broken, how and who can fix it for you? Is that the same person who fixes fans and electricity? Is he qualified to fix medical devices? Therefore, there is no quality in human resources and job description”. [M]
	Lack of competent and qualified employees in quality	There is a shortage in quality and quantity of qualified and well-trained staff, this reason causes many predicaments in the workplace. Yemen has no standards and criteria in quality, also medical administrators have no guidelines to follow”. [A2]
	Insufficient health personnel.	“We have too much work but there is a shortage of workers, which impedes providing quality. Also, we don’t have qualified managers promoting the high-quality work”. [A2]
	Inadequate training for healthcare professionals.	“There are no training courses and workshops for the hospital staff and healthcare professionals on quality systems and accreditation”. [A3]
Financial constraints	High cost of quality	“We lack financial support locally and internationally; any hospital can’t obtain accreditation and attain quality without this support, obtaining quality is costly”. [M]
	Insufficient budget allocations	“Our hospitals suffered from insufficient budgets, which reflect on quality systems and accreditation”. [H]
	Inadequate incentives.	“There are no monetary incentives or moral incentives for healthcare professionals to work extra hours for accreditation and quality”. [N]
	Low wages	“The medical staff had poor financial compensation, which led many of them to engage in external work”. [F]
Lack of awareness of quality systems and accreditation	Low awareness of quality among healthcare professionals	“There is inadequate awareness among medical staff or administration regarding raising the level of quality in their healthcare institutions. Even so, they don’t recognize the way of raising the quality, for example, the essential tools that should be available within their department and the protective measures and guidelines. In the X-ray room, the X-ray technician does not wear the proper uniform or apron to protect himself”. [M]
	Insufficient knowledge of quality	“Most of the administration and medical staff do not understand the concept of quality due to lack of training in the workplace. Also, special training should be provided to other workers, such as the sanitation workers”. [A2]
		“We have weak curriculums in the institutes and universities that don’t focus on updating knowledge and evidence-based practice to make graduates succeed in their future fields and work effectively. Healthcare institutions don’t take care of the quality of the graduates due to a shortage of medical staff. Also, these institutions lacked awareness of the quality systems and their importance”. [H]
	Low acceptance of quality	“If the institutions hold workshops and training about accreditation and quality systems, the attendance of healthcare professionals is low, which reflects that they don’t accept these concepts”. [A3]
Lack of monitoring of healthcare professionals	Poor monitoring of health records	“We do not have disciplinary boards tracking problems and giving penalties and following up complaints and problems”. [A2]
	Poor monitoring of healthcare providers’ performance	There is no ongoing monitoring of medical staff, which causes a lack of quality. The quality department should be responsible for monitoring quality standards within the hospital, it is the eye of the manager”. [B]
		“There is weak supervision of new workers and mentorship from senior workers. There are many malpractices and inappropriate penalties for faults in the health field”. [N]

Strategies for Improving the application of quality standards

Strategies to improve quality standards encompassed two themes: improving the quality system in healthcare institutions and promoting quality standards among healthcare professionals. Table 2 explains these themes with sub-themes and quotations.

**Table 2:** Strategies for improving the application of quality standards O, A1, F, A2, M, B indicate the different participants

Theme	Sub-theme	Supporting quotation
Improving quality systems in healthcare institutions	Establishing a specialized quality department with clear standards.	“There should be a quality department in each hospital to implement the quality standards properly.’ [A1]
	Ongoing enhancements of quality work environment	“We should establish quality measures in the application of regulations in healthcare institutions and focus on the quality standards in work.”. [O]
		"It is necessary to implement quality standards in all working situations." [A1]
	Reconstructed quality systems.	“We should establish high-quality infrastructure and quantity in the field of work. Additionally, equipment to provide the necessary services should be available and accessible”. [F]
	Allocating sufficient budgets based on policies	“Also, providing adequate budgets for hospitals and health facilities could be effective.” [O]
	Planning and applying monitoring quality systems.	“We need to create an organizational structure for management. It is necessary to be followed up by high-level authorities”. [B]
		“Evaluation and follow-up in the workplace from administrators should be enhanced”. [A1]
	Adequate health care professionals in quality standards.	“We need to hire an adequate number of health personnel. Also, scientific research to raise the level of quality standards should be encouraged”. [A2]
Promoting quality standards among health staff	Consistent training programs and workshops.	“We can enhance quality standards by providing continuous training and workshops about quality to produce qualified and competent medical staff in all medical departments.” [O]
		“Educational curriculum in institutes and universities should be reviewed and modified according to up-to-date studies”. [M]
	Staff motivation and incentives.	“We should recognize the importance of motivating employees with job loyalties through awareness of the profession from the religious aspect”. Also, rewarding the staff in the case of satisfactory work.” [B]
		“It is essential to provide required courses to raise quality standards in the workplace. Moral and monetary incentives and rewards for the staff (workers and medical) should be provided. Also, elevating awareness in all workers should be implemented”. [A1]

## Discussion

This study revealed that lack of proper infrastructure, financial constraints, lack of awareness of quality systems and accreditation, and lack of monitoring of healthcare professionals hinder the implementation of accreditation.

All the participants mentioned the paucity of unique quality systems in some healthcare establishments and poor infrastructure and quality standards in hospitals, specifically in rural areas. A lack of competent personnel in quality and trained medical staff to provide quality care was also necessary. These establishments did not conform to any form of authority in implementing quality standards. Furthermore, inadequate medical equipment and management approach to implementing quality standards, incompetent health personnel in some hospitals, and insufficient training for healthcare professionals led to poor quality standards and systems. Hospitals with poor qualifications should apply accreditation standards, which was one of the key elements proven in this study. This finding is proven by studies on the weak healthcare system by Al Kuwaiti and Al Muhanna [22], Binagwaho et al. [23], Janati et al. [7], and Vali et al. [24]. This study result is expected due to the situation of the current healthcare condition in war-ridden Yemen.

Eight of the participants reported the high cost of quality as the biggest challenge in complying with quality systems apart from the lack of financial budgets to apply quality standards, inadequate incentives, and low wages. This study revealed poor financial resources and budgets as one of the key obstacles to implementing quality systems in Yemeni hospitals in line with past works [8,9,12,25,26]. This finding also corroborates the current situation in Yemen, which has led some managers to perceive accreditation as unnecessary [22].

All the participants stated that low awareness of quality among healthcare providers in hospitals and inadequate knowledge of quality-related notions hampered the obtainment of accreditation and quality systems. This finding was similar to that of past studies [27], where managers’ low awareness of quality, accreditation, and commitment hindered its application [28].

From the perspective of healthcare workers, poor monitoring and follow-up systems for individual and organizational performance deterred the application of accreditation programs. This finding corresponds with that of the studies by Ng et al. [6], and Zineldin [29].

The healthcare professionals in this study indicated the lack of awareness of accreditation and quality and the paucity of a holistic organizational environment that trains employees and buildings to implement quality and accreditation systems as key challenges in the hospitals of the Hadhramaut region. The Ministry of Health should facilitate the preparation of healthcare facilities, specifically hospitals, to apply quality and accreditation standards by reconstructing buildings, providing the necessary supplies, and preparing and training health leaders and hospital managers to support the implementation of quality and accreditation programs in these facilities. The current conflicts in Yemen have proved detrimental to the country’s health sector and patients’ safety. Hence, local and global WHO-led donors should periodically allocate budgets to strengthen the infrastructure of Yemeni hospitals. Private hospital owners’ and government hospital directors’ awareness and interest would also determine the implementation of quality standards and hospital accreditation.

To the best of our knowledge, this is the first study in Yemen that discussed accreditation in healthcare institutions. One limitation of this study is the small sample size because of which the results may not be generalizable to all healthcare professionals in Yemen. 

## Conclusions

This qualitative study explored the challenges and strategies in implementing accreditation systems in Yemeni (Hadhramaut) hospitals via focus group discussion. As a result, the themes associated with the challenges underlying accreditation included lack of proper infrastructure, financial constraints, lack of awareness of the quality system and accreditation, and lack of monitoring of healthcare professionals. Additionally, the strategies to improve quality standards included improving the quality system in healthcare institutions and promoting quality standards among healthcare professionals. Thus, policymakers should consider these barriers and strategies to develop policies and strategies for enhancing quality in healthcare institutions.

Also, future studies are recommended to evaluate accreditation and quality programs in healthcare institutions. Thus, policymakers should consider these barriers and strategies to develop policies and strategies for enhancing quality in healthcare institutions. Future studies are also recommended to include healthcare professionals from different regions of Yemen to evaluate accreditation and quality programs in healthcare institutions.
